# Brief Report: A Comparison of the Preference for Viewing Social and Non-social Movies in Typical and Autistic Adolescents

**DOI:** 10.1007/s10803-016-2974-3

**Published:** 2016-11-23

**Authors:** Indu Dubey, Danielle Ropar, Antonia F. de C. Hamilton

**Affiliations:** 1 School of Psychology, University of Nottingham, University Park, Nottingham, NG7 2RD UK; 2 Institute of Cognitive Neuroscience, University College London, Alexandra House, 17 Queen Square, London, WC1N 3AR UK

**Keywords:** Choose-a-Movie (CAM) task, Social preference, Social motivation, Autism spectrum conditions (ASC), Adolescents

## Abstract

The recently proposed Social Motivation theory (Chevallier et al., Trends in cognitive sciences 16(4):231–239, 2012) suggests that social difficulties in Autism Spectrum Condition (ASC) might be caused by a difference in the motivation to engage with other people. Here we compared adolescents with (N = 31) and without (N = 37) ASC on the Choose-a-Movie paradigm that measures the social seeking. The results showed a preference for viewing objects over smiling faces in ASC, which is in line with the theory of low social motivation. However, typical adolescents did not show any stimuli preferences, raising questions about developmental changes in social motivation. Age was found to play a significant role in moderating the choice behaviour of the participants. We discuss the implications of these findings in detail.

**Electronic supplementary material:**

The online version of this article (doi:10.1007/s10803-016-2974-3) contains supplementary material, which is available to authorized users.

## Introduction

Social difficulties are a defining feature of autism spectrum conditions (ASC). Recently it has been suggested that social symptoms of ASC may stem from a motivational deficit (Chevallier et al. 2012). This social motivation theory proposes that social interactions are inherently rewarding and motivating for most typically developing people but this might not be true for people with ASC. Here we test if adolescents with ASC differ in their preference for viewing social/non-social movies, as a way to evaluate the social motivation theory.

Observations of the behaviour of children with autism in natural settings suggest fewer friendships and reciprocity (Chamberlain et al. 2007) and adults with autism also report less desire for friendship (Baron-Cohen and Wheelwright 2003). However, quantifying motivation in the lab is not always straightforward. Chevallier et al. (2012) distinguish three domains of social motivation—orienting towards people; seeking out people and maintaining social relationships over the long term. We focus here on measuring the propensity to seek out others or find interaction with others rewarding, rather than just visual orienting towards particular stimuli. Approach-avoidance tasks have been used previously to understand the motivation to avoid fearful stimuli such as spiders (Rinck and Becker 2007) or social stimuli in people with social anxiety (Heuer et al. 2007). In this task, target stimuli are shown on a computer screen and participants can either pull the item towards themselves (making it larger) or push the item away (making it smaller) using the joystick, giving an estimate of their approach and avoidance preferences for the stimuli. Studies of social motivation in ASC using this type of task have shown some mixed results, including a higher approach tendency for all the stimuli (face as well as landscapes) in people with ASC (Deckers et al. 2014) or greater approach for only the stimuli with higher incentive value for ASC (Silva et al. 2015). Ewing et al. (2013) also failed to find evidence of lower social preference in adolescents with ASC, and reported high preference for non-social stimuli in both ASC and typical groups. Similarly, Watson et al. (2015) found that participants with ASC would accept lower monetary reward to look at high autism interest objects, while they were not different from typical groups for their preference for social or non-social objects.

There are several possible reasons for the mixed results reported above. One is the substantial heterogeneity in the symptom profiles and cognitive abilities within ASC (Freeth et al. 2011; McPartland et al. 2011). The other possible reason could be the difference in the tasks used and the age groups of the participants taken in these studies. There is increasing evidence that motivation to engage with different social groups changes over the course of typical adolescence (Foulkes and Blakemore 2016), which could impact on the behaviour of both the autism and the typical control sample in the studies described above.

Here we use a simpler task, called Choose-A-Movie, which can measure social motivation in adults with ASC (Dubey et al. 2015). The aim of the present study is to test if this autistic difference in social motivation is also present in adolescents, and to gain a deeper understanding of the development of social preference. Based on the theory of reduced social motivation and the previous findings from the original version of CAM paradigm with adults with ASC, it was expected that the adolescents with ASC would show reduced preference for social stimuli.

## Method

### Participants

79 adolescents with and without autism between the ages of 11.17–18.50 years took part in this study. To create two well-matched groups for optimum data analysis, data from 11 participants were excluded from the analyses reported below (full data is reported in supplementary material Section 6). The final group of typically developing participants (TD) consisted of 37 adolescents, none of which had a clinical diagnosis of any condition as confirmed by their parent or caregiver. The final group of participants with ASC consisted of 31 adolescents all of whom had an independent diagnosis of ASC by a Paediatrician, Psychiatrist, Psychologist, or other trained clinician (details of diagnosis are given in supplementary material Section 1) (Table 1).

**Table 1:** Description of the matched groups

	ASC group n = 31 M (±SD) range	Typical group n = 37 M (±SD) range	Difference
M:F	26:5	34:3	
Age (years)	14.22 (±1.84) 11.17–18.50	13.74 (±1.12) 11.33–16.16	*t* (66) = 1.355, *p* = 0.180
BPVS*	131.23 (±25.79) 75–165	136.70 (±9.65) 109–155	*t* (66) = −1.197, *p* = 0.236
RPM*	38.26 (±8.96) 16–51	39 (±7.25) 23–51	*t* (66) = −0.377, *p* = 0.707
SRS (score range 0–195)	n = 25, 113.24 (±25.22) 48–152		
SAS (score range 0–40)	n = 27, 7.70 (±5.74) 1–23		

*Raw score were used for BPVS and RPM

### The Choose-A-Movie (CAM) Paradigm

The CAM paradigm measures social motivation by giving participants a choice of which movie to watch under conditions where different levels of effort are required to view each movie (See Dubey et al. 2015). The logic of the task is that participants see two boxes containing movies from two categories, and must choose which box to open. For example, the orange spotty box contains social movies and the pink patterned box has object movies (or vice versa for counterbalancing). Effort is applied by placing locks on the boxes, which must be opened with an action followed by a delay which puts a small but noticeable time delay on the choice to open a box with more locks.

In the adult version, three different stimulus categories were tested (direct gaze videos, averted gaze videos and object videos), requiring 180 trials to give enough binary choices between each pair of stimuli. Here, we tested just two stimulus categories (direct gaze videos and object videos) and were able to reduce the number of trials to 60. The videos used here were identical to Dubey et al. (2015), that is, the direct gaze video set comprised 10 movies of smiling adults who look directly at the camera for 3 s. The object movies show pairs of household objects on a slowly moving turntable for 3 s.

**Fig. 1:**
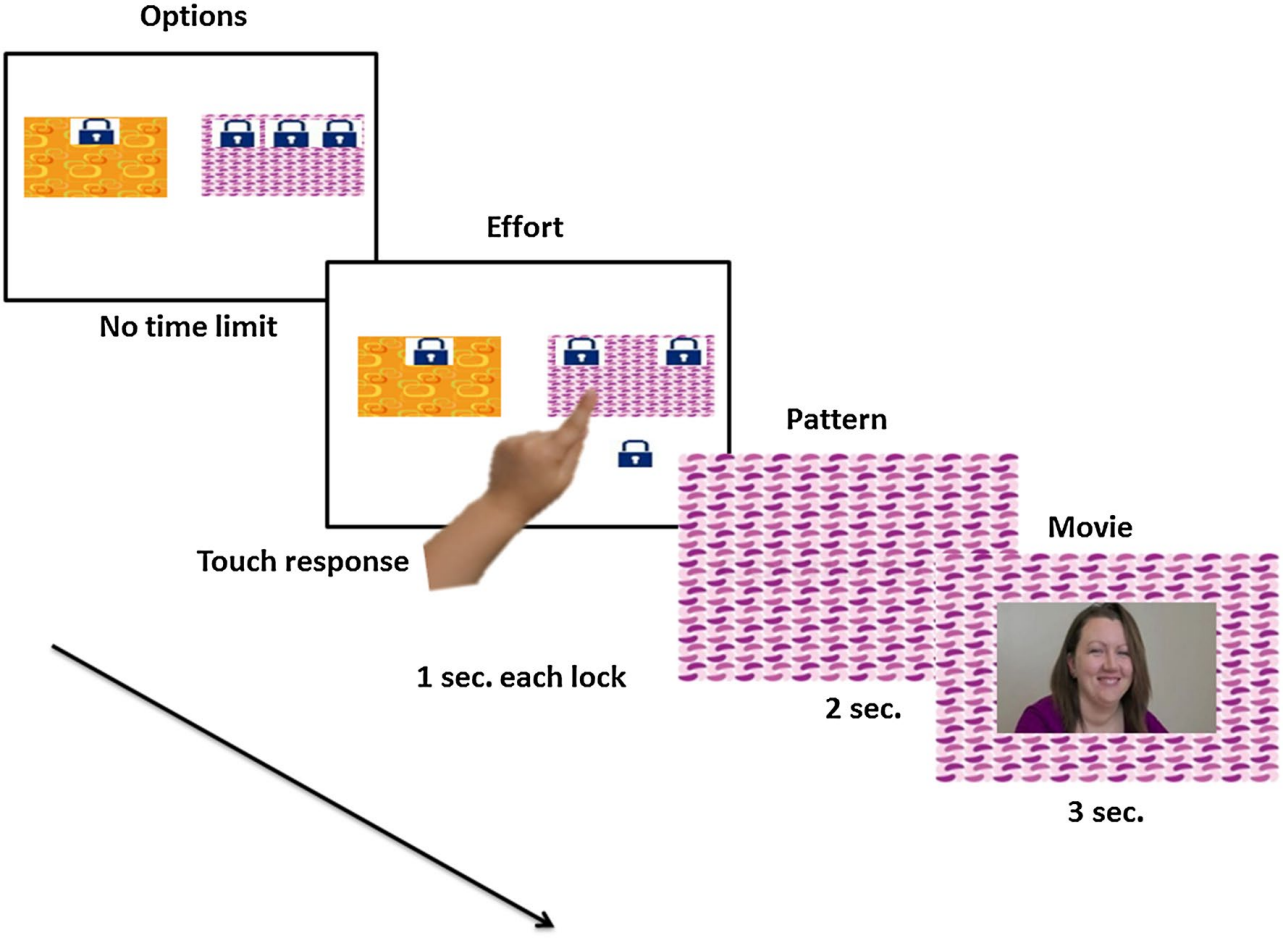
Example of an experimental choice trial in which participant is presented with *box* with object movies with one lock and *box* with social movies with three locks. Participant here touched the *pink* (social) *box* to open, making relatively higher effort (+2 locks) to look at a movie from his/her preferred stimuli category. (Color figure online)

We implemented the CAM paradigm in almost exactly the same way as Dubey et al. First, participants learnt the association between two coloured boxes and the two sets of stimuli movies (e.g. pink box has object movies) by seeing each coloured box together with six sample still images from that movie category for 3 s. Then participants completed 10 learning trials, where one box with one lock was present on the screen. The participant touched the lock to remove it. Once the lock was removed the participant watched one of the linked movies. This gave participants a chance to become familiar with the two types of boxes, touching the screen to remove locks, and seeing movies form the different categories. After learning was complete, participants completed 60 choice trials which allowed for the collection of the experimental data. On each trial, participants saw two boxes on the screen with between 1 and 3 locks on each box. Participants chose any one box to open and remove the locks by touching them. When all the locks on one box were removed, that box opened to show a movie from the associated set of stimuli (Fig. 1). The only differences between this procedure and Dubey et al. 2015 were (a) using 2 movie categories rather than 3 (b) using a touch screen for responses rather than a keyboard and (c) a clearer learning phase.

The mapping between the box pattern and the movie category was constant for each participant and counterbalanced across participants. The 60 choice trials included 24 trials which had 3 locks on one box and 1 on the other; 12 with 2 locks on one box with 1 on the other, 12 with 3 locks on one box with 2 on the other, and 12 having equal numbers of locks on each box. The boxes with the larger number of locks were pseudo-randomly assigned to the left or right side of the screen with equal probability for appearing on both sides. On each trial, a participant could choose to open the box with fewer locks (fewer touches and quicker) or the box with more locks (more touches and slower). Thus, participants were encouraged to make a trade-off between the effort required to open the box and their preference for a particular movie category.

### Procedure

Participants whose parents/caretakers had consented, took part in the study in several one-to-one sessions a quiet room in their school with little distraction. During these sessions, they completed the CAM task using MATLAB with Cogent toolbox on 12 × 6.5-inch screen of a Samsung Ultrabook (touch screen). They also completed measures of verbal, non-verbal intelligence and social abilities (see supplementary material Section 2). The sequence of administration of these tasks could vary depending on the schedule of the school and availability of the participant. The participants were given breaks between the tasks (when needed).

### Data Analysis

Our primary outcome measure is the choices participants make in the CAM task, which we code as a 1 for ‘chose video on the left’ and 0 for ‘chose video on the right’. We characterise each trial according to the video stimulus presented on the left (social or non-social) and the relative effort required to choose the video on the left (for example, a trial with 3 locks on the left box and 1 on the right box would be coded with Effort of +2; a trial with 1 lock on the left box and 2 on the right would be coded with Effort of −1). These two trial factors (Stimulus and Effort) were used in a mixed model logistic regression with the participant-level factors of diagnostic group, age, BPVS score and RPM score to predict the choices made on each trial.

## Results

To understand the predictive value of effort, stimuli, groups, and their interaction on the choice made by the participants, mixed model logistic regression analysis was used. Here only main results are discussed and all the other results are presented in Table 2. Overall results suggest that the choices of the participants were influenced primarily by the effort required (Wald χ^2^ = 45.317, *p* < 0.0001), and marginally by the stimuli (Wald χ^2^ = 3.741, *p* = 0.053).

**Table 2:** Results from logistic regression: factors influencing participants’ decision to choose stimuli presented on left side

	All participants (Wald χ^2^, *p*)
Effort	45.317, <0.0001
Stimuli	3.741, 0.053
Groups	1.863, 0.172
Stimuli × effort	3.843, 0.428
Stimuli × group	2.103, 0.147
Effort × group	7.681, 0.104
Stimuli × effort × group	5.525, 0.238

**Fig. 2:**
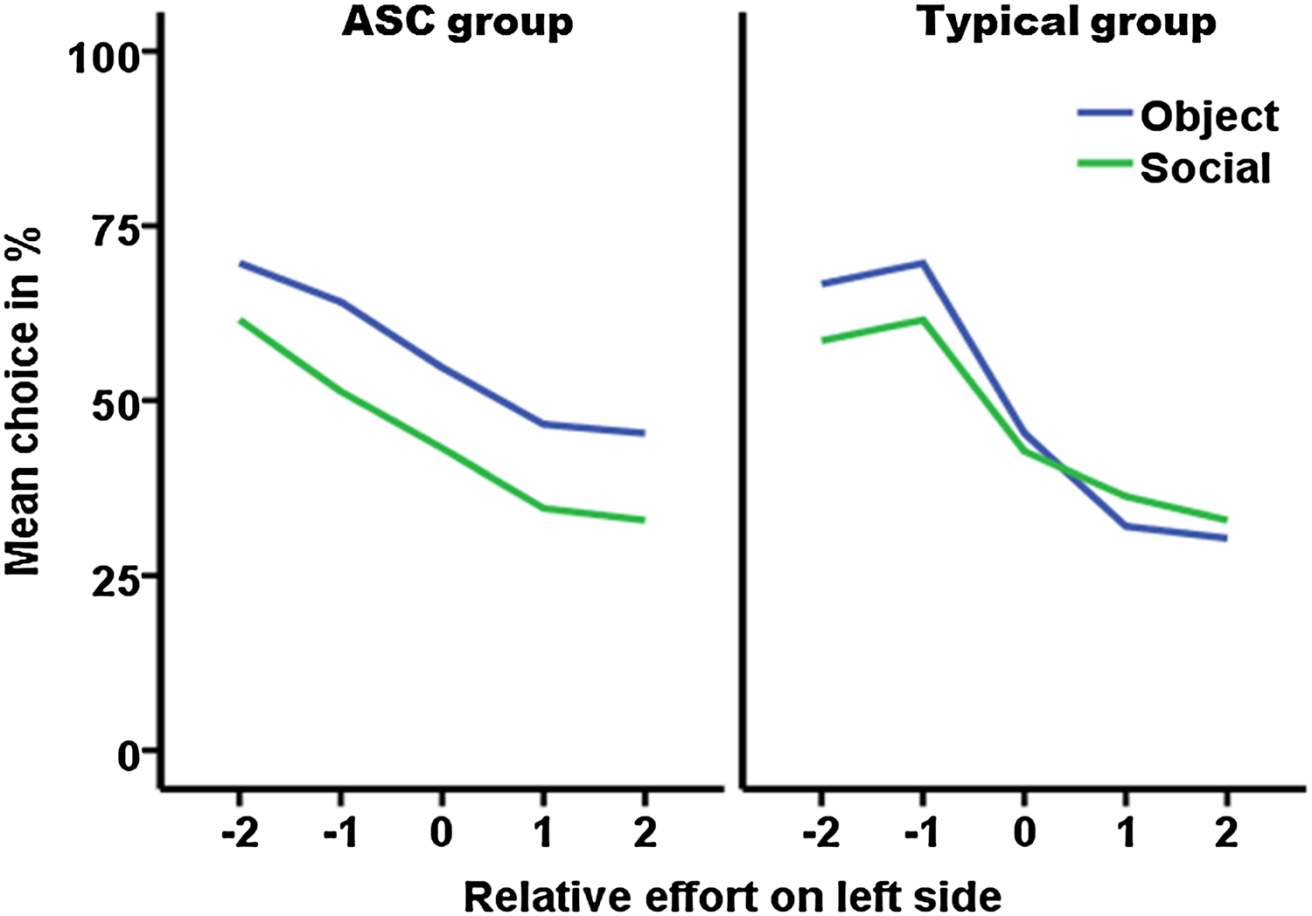
Figure shows mean percentage (*Y axes*) of times participants from each group chose social (*green line*) or non-social (*blue line*) stimuli when presented on *left side* with relative lock difference (effort) of −2 to +2 (*X axes*). The difference between the two lines shows the extent of preference for one stimulus over the other (larger the difference stronger the preference). A −2 relative lock difference indicates that there was 1 lock on the *left side* and 3 on the *right side*. (Color figure online)

To explore the choice patterns of each group, the logistic regression was performed separately for each participant group. The results showed that the choices made by the ASC group were significantly influenced by the effort (Wald χ^2^ = 22.660, *p* < 0.0001) and stimuli (Wald χ^2^ = 4.567, *p* = 0.033), but not by their interaction (Wald χ^2^ = 1.418, *p* = 0.841). On the other hand, choices of the matched typical group were significantly influenced by the effort (Wald χ^2^ = 26.018, *p* < 0.0001), and an interaction between effort and stimuli (Wald χ^2^ = 10.388, *p* = 0.034) but not by stimuli (Wald χ^2^ = 0.157, *p* = 0.692). The pattern of choices for each group are illustrated in Fig. 2. The data in the left panel show that the ASC group have a clear preference for the object videos over the social videos, indicated by the green line above the blue line, but also took the Effort factor into account as indicated by the downward slope of the lines. The data in the right panel show no clear stimuli preference in the TD group. Their preference for non-social stimuli was higher when stimuli were presented with low effort and the opposite pattern was observed on high effort trials (Fig. 2); effort influenced choices in both cases.

Further analyses of the basic preference for stimuli irrespective of effort levels; effect of age; and effect of intelligence on choice behaviour of both the groups are presented in supplementary material Section 3, 4, and 5 respectively. We also report the relationship between basic preference and social behaviour as scored on the SRS in supplementary Section 3.

## Discussion

The aim of this study was to explore social motivation in adolescents with and without ASC. Results from this study suggest that participants with ASC prefer non-social movies of household objects over social movies of smiling people, however, they are not completely indifferent to the effort involved in their choices. When their preferred non-social stimuli were presented with higher level of effort (more locks than the social stimuli) than the alternative, they trade-off their preference for the less effortful choice.

These results are comparable to several previous studies. In the previous study using CAM paradigm with adults with ASC (Dubey et al. 2015), we also found a people with ASC prefer movies of non-social items. Similar findings are presented by Ewing et al. (2013), who reported that adolescents with ASC preferred looking at non-social stimuli more than social stimuli. Like the present study they found no difference between the ASC and the matched typical group for their approach to social or non-social stimuli. A comparable finding is also reported by Sasson et al. (2008), who found that both ASC and matched typical adolescents prefer to explore non-social stimuli more than social. They also reported that the participants with ASC spent less time looking at social images when presented against high autism interest images than low autism interest images. Lower reward activation for social stimuli in ASC is also reported on a Social Incentive Delay task used with brain imaging studies (Damiano et al. 2015; Delmonte et al. 2012). Overall, these results seem to support the theory of reduced social motivation in people with ASC. Alternative explanations concerning the role of anxiety in autism are discussed in the supplementary material Section 7.

It is known that the changes in the hormones and brain development alter the value of social stimuli during adolescence years in typical people (Blakemore 2010; Nelson et al. 2005). In light of the literature, we expected that typical adolescents would have heightened sensitivity for the social stimuli resulting in higher motivation to seek it (Foulkes and Blakemore 2016). Counterintuitively, the typical adolescents on our study did not show the same social preference as seen previously in typical adults (Dubey et al. 2015). These findings are similar to Ewing et al. (2013) and Sasson et al. (2008), who also reported stronger preference for non-social stimuli in the typical adolescent participants. One reason for the lack of preference for social stimuli might be the nature of the stimuli used here. The social stimuli in the present study were faces of adults (actors aged approx. 22–32 years), which might be less appealing to this participant group than the faces of other adolescents (peers). And as the heightened sensitivity for social stimuli seen in the adolescents is generally limited to the peer group, they may not be motivated to seek social stimuli with adult faces (Knoll et al. 2015). An alternative explanation of lack of social preference in adolescents due to major developmental changes in brain structure at this age is discussed in the supplementary material (Section 8).

### Limitations and Future Directions

Like any other laboratory based tool, CAM might have difficulties of generalizability of findings to more naturalistic situations. Even though the CAM task has been used previously to measure social motivation in adults, in absence of any other standard measure of social motivation, it is difficult to establish the construct validity of the tool. Current data does not show a relationship between the SRS-social motivation subscale and the social preference score on this task (Supplementary Section 3), but a larger sample size would be needed to test this fully. It will be important in future to compare the social motivation of people as evaluated on CAM with other behavioural paradigms and clinical observations.

## Conclusion

To summarise, the present study shows that adolescents with ASC show low social preference on the CAM paradigm, supporting the theory of reduced social motivation in ASC. However, important developmental changes may reduce the social preference in typical groups. We suggest it is important to understand the development of social motivation and its possible relationship to social anxiety in participants with and without ASC.

## Electronic supplementary material

Below is the link to the electronic supplementary material.

Supplementary material 1 (DOCX 283 KB)
