# Supplementary material for: Brief Report: A Comparison of the Preference for Viewing Social and Non-social Movies in Typical and Autistic Adolescents
Source: J Autism Dev Disord. 2016 Nov 23;47(2):514–9. doi: 10.1007/s10803-016-2974-3 (PMC5309295; doi:10.1007/s10803-016-2974-3)
Supplement: Supplementary file 1 — Supplementary material 1 (DOCX 283 KB) [file 10803_2016_2974_MOESM1_ESM.docx]

**Supplementary Material**

**Supplementary Section 1**

**Methods**

**Diagnostic information of the participants with Autism Spectrum Conditions (ASC)**

Diagnostic information for the participants with ASC was obtained through school records and the information provided by the individual’s parent/guardian. Most participants received an independent diagnosis of ASC by a Paediatrician, Psychiatrist, Psychologist, or other trained clinician before the age of 10 years. The participants were included in the study irrespective of the sub-category of the diagnosis within ASC and associated conditions. Thirteen of these participants had no other diagnoses, the remaining participants had one or more additional conditions, including Attention Deficit Hyperactive Disorder (n=9), learning disability (n=7), dyspraxia (n=6), anxiety disorder (n=3) and other conditions (n=13) such as Tourette syndrome, and epilepsy. Details about additional diagnoses were not available for five participants. We did not do any additional diagnostic evaluation of the participants but their current social functioning, which is the most important aspect of autism for the current study was evaluated using Social Responsiveness Scale - SRS (Constantino and Gruber 2005). The SRS has high sensitivities (.74 to .80) and specificities (.69 to 1.00) for autism diagnosis (Bölte et al. 2011). It is also known to have good correlation with Autism Diagnostic Observation Schedule - ADOS (Lord et al. 2000) (*r* = .35 with the social domain) and Autism Diagnostic Interview-revised- ADI-R (Lord et al. 1994) (*r* = .45 with the social domain) (Bölte et al. 2011).

**Supplementary Section 2**

**Tools**

Participants completed two tests of intelligence the British Picture Vocabulary Scale (3^rd^) - BPVS III (Dunn et al. 1997), which is a standardised tool for the evaluation of verbal intelligence in people between 3-17 years. The Raven’s Progressive Matrices - RPM (Raven et al. 2003), which is a multiple choice test of abstract reasoning, used for the evaluation of non-verbal intelligence.

The parents/caretakers of all participants were asked to complete two questionnaires by post: the Social Responsiveness Scale - SRS (Constantino and Gruber 2005) and the Social Aptitude Scale - SAS (Liddle et al. 2009). The SRS is a 65 item scale completed by the parent or guardian of the person which takes about 15 minutes to complete and assesses a person’s level of social functioning. The scale yields score between 0-195 with higher scores indicating greater social difficulties. A score of ⩾75 is accepted as the cutoff to identify people with ASC (Constantino and Gruber 2005). The SAS is a 10 item scale to evaluate social functioning of the person. Scores can range between 0-40 with lower numbers indicating a higher level of social difficulties. The SAS has a cut-off score of 16 or less for identifying individuals with possible ASC. The SRS and SAS both have very good sensitivity and specificity as screening tools (Aldridge et al. 2012; Liddle et al. 2009) and were used in the present study to evaluate the current level of social difficulties in these participants. Due to poor rates of return of the questionnaires from parents of typical participants, we report only the data from the ASC group to allow comparison of levels of social ability in this group to other studies.

**Results**

**Supplementary Section 3**

**Basic preference**

To understand the basic stimuli preference for the participants, we first collated all the trials irrespective of the effort levels and calculated percentage of trials on which participants chose social or non-social stimuli. For each group separately, we compared their preference to the chance level of 50%. The results suggested that participants with ASC had a significantly lower preference for the social stimuli than chance value (*t* (30) = -2.10, *p* = 0.044) (Supplementary Fig 1), but the TD group had no preference (*t* (36) = -0.03, *p* = 0.974). We also compared two groups to each other, and found no significant difference between the groups (*t* (66) = -1.598, *p* = 0.114). As the basic analysis does not take in to account the role of effort on the decision making, we further performed the logistic regression analysis, explained below.


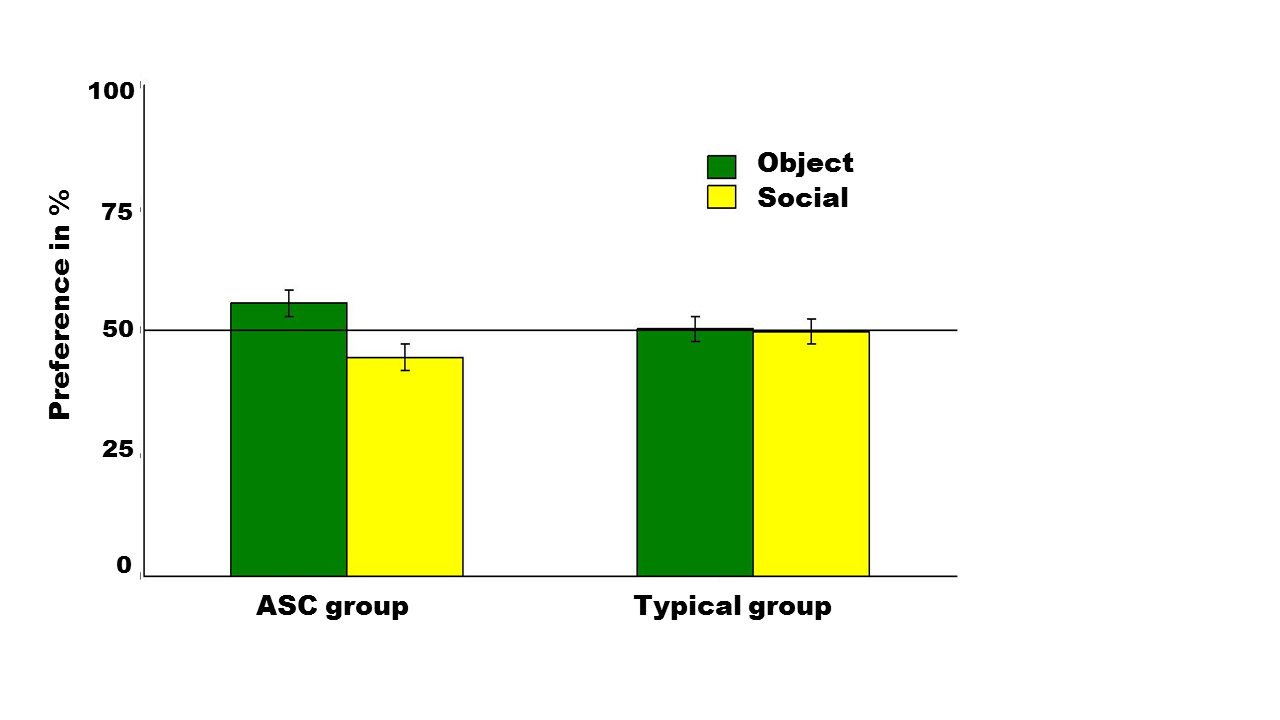


**Supplementary Figure 1:** Figure shows mean percentage of preference for social and non-social stimuli in two groups.

**Relation between social preference and social motivation subscale of Social Responsiveness Scale (SRS)**

We compared the social preference (collapsed over all the cost conditions) to the score on social motivation subscale of SRS (for group with autism). The results show that the two score do not have a significant correlation (*r*= .048, *p* = .821). However, it must be noted that social motivation subscale has only 11 items that measure both social approach behaviour as well as social anxiety.

**Supplementary Section 4**

**Effect of Age on choice**

**
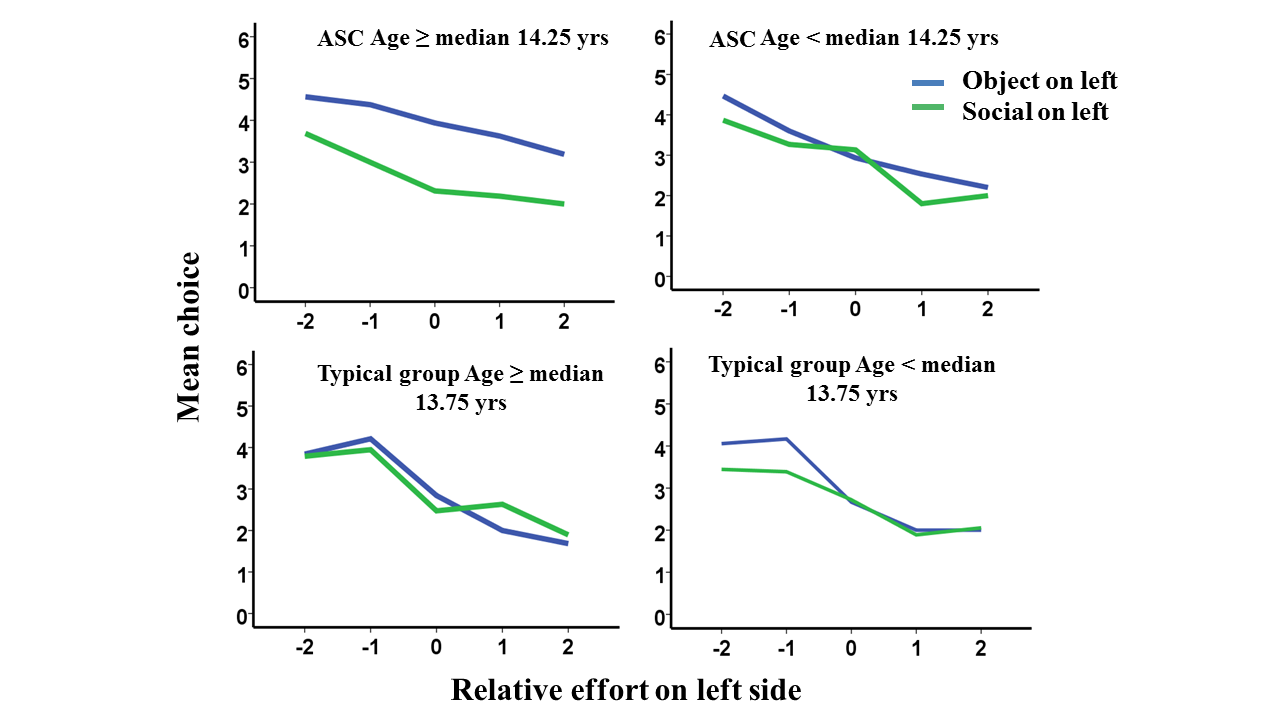
**To illustrate the role of age in the choice behaviour of the participants, we plotted the group results with a median split (Supplementary Fig 2). As the figure shows, older ASC participants have a stronger preference for non-social stimuli than younger ASC participants, while age does not relate to preferences in this typical sample. It indicates that age might influence how participants with ASC respond to the choice between social and non-social stimuli.

**Supplementary Figure 2:** Figure shows mean percentage (Y axes) of times participants from each subgroup: medial split of age for two groups, chose social (green line) or non-social (blue line) stimuli when presented on left side with relative lock difference (effort).

**Supplementary Section 5**

**Effect of intelligence on choice behaviour**

To explore the role of age and intelligence on choice behaviour, we conducted three further logistic regressions with each of these factors as an additional regressor. The results from these are presented below in supplementary table 1. We highlight the finding that effort consistently influences choices, and age might moderate how stimuli and effort influence the choice behaviour of the participants (stimuli by group by age interaction Wald χ^2^ = 4.009, *p* = 0.045).

**Supplementary table 1:** Logistic regression with other factors namely age, non-verbal intelligence (RPM), and verbal intelligence.

| **Factors** | | **Age** | | **RPM** | | **BPVS** | |
| --- | --- | --- | --- | --- | --- | --- | --- |
| Effort | 9.953,  *p* = 0.041 | | 13.572,  *p* = 0.009 | | 6.483,  *p* = 0.166 | |  |
| Stimuli | 0.462,  *p* = 0.497 | | 2.157,  *p* = 0.142 | | 0.002,  *p* = 0.966 | |  |
| Group | 1.367,  *p* = 0.242 | | 0.005,  *p* = 0.945 | | 7.898,  *p* = 0.005 | |  |
| Stimulus X effort | 4.091,  *p* = 0.394 | | 3.594,  *p* = 0.464 | | 1.892,  *p* = 0.756 | |  |
| Stimuli X group | 3.393,  *p* = 0.065 | | 1.861,  *p* = 0.172 | | 0.388,  *p* = 0.534 | |  |
| Effort X group | 0.763,  *p* = 0.943 | | 2.169,  *p* = 0.705 | | 3.923,  *p* = 0.416 | |  |
| Effort X stimuli X group | 1.367,  *p* = 0.065 | | 1.210,  *p* = 0.876 | | 0.251,  *p* = 0.993 | |  |
| Effort X *factor* | 6.616,  *p* = 0.158 | | 7.854,  *p* = 0.097 | | 4.041,  *p* = 0.400 | |  |
| Stimuli X  *factor* | 0.279,  *p* = 0.597 | | 1.228,  *p* = 0.268 | | 0.073,  *p* = 0.787 | |  |
| Effort X stimuli X  *factor* | 4.445,  *p* = 0.349 | | 3.246,  *p* = 0.518 | | 1.591,  *p* = 0.810 | |  |
| Stimuli X group X  *factor* | 4.009,  *p* = 0.045 | | 2.678,  *p* = 0.101 | | 0.623,  *p* = 0.430 | |  |
| Effort X group X  *factor* | 1.411,  *p* = 0.842 | | 1.052,  *p* = 0.902 | | 3.954,  *p* = 0.412 | |  |
| Effort X stimuli X group X  *factor* | 1.318,  *p* = 0.858 | | 2.302,  *p* = 0.680 | | 0.220,  *p* = 0.994 | |  |

**Supplementary Section 6**

**Analysis using full data sets not matched for intelligence**

The data was originally collected form 39 ASC and 40 Matched control participants. As the two groups did not match on BPVS, 8 ASC participants and 3 from the typical group were excluded from the main analysis. In this section we present a separate analysis done on full dataset. In this analysis The missing values on measures of BPVS, RPM, SRS, SAS and age were replaced by the group means. The description of the unmatched groups is given in supplementary tables 2 and the findings of the logistic regression for the unmatched groups is presented in supplementary tables 3. The main results from the logistic regression for unmatched groups are largely same as the matched groups.

**Supplementary table 2:** Group description for all the participants. Here *N* represents the available data for the subgroups. Raw scores were used for BPVS, RPM, SRS and SAS.

|  | ASD group | Control Group | Difference |
| --- | --- | --- | --- |
| M : F | *N*=39, ratio = 34:5 | *N* =40, ratio = 36:4 |  |
| Age  (Years) | *N* =38, *M* =14.11,  *SD* ±1.92 | *N* =38, *M* =13.73,  *SD* ±1.11 | *t* (59.22) = 1.04, *p* = 0.303 |
| BPVS | *N* =35, *M* =127.29,  *SD* ±27.94 | *N* =40, *M* =137.35,  *SD* ±10.23 | *t* (41.94) = -2.12, *p* = 0.037 |
| RPM | *N* =34, *M* = 37.94,  *SD* ±9.24 | *N* =39, *M* =39.46,  *SD* ±7.34 | *t* (71) = -0.78, *p* = 0.436 |

**Supplementary table 3:** Results from logistic regression for the matched groups: factors influencing participants' decision to choose stimuli presented on left side.

|  | **Matched participants (Wald χ ^2^ , *p*)** |
| --- | --- |
| Effort | 47.929, *p* < .0001 |
| Stimuli | 3.674, *p* = 0.055 |
| Groups | 2.038, *p* = 0.153 |
| Stimuli X effort | 5.898, *p* = 0.207 |
| Stimuli X group | 1.396, *p* = 0.237 |
| Effort X group | 8.276, *p* = 0.082 |
| Stimuli X effort X group | 6.722, *p* = 0.151 |

**Discussion**

**Supplementary Section 7**

**Effect of age on social seeking in ASC**

A closer look at the data suggest that age is also an important factor in understanding social motivation in adolescents with ASC. We found an interaction between Group, Age and Stimuli, and an illustration of the age effect suggests that the older participants with ASC (over 14.25 years, n=16) showed a strong preference for the non-social stimuli, but the younger participants with ASC (under 14.25 years, n=15) showed no preference. It is worth noting here that the behaviour of the older ASC sample is consistent with the adult ASC sample tested in Dubey et al 2015, but the lack of a non-social preference in the younger ASC group is surprising. This implies that social motivation changes over the 11 to18 age range in participants with ASC, and that younger participants may not show diminished social motivation in the same way that participants over the age of 14.25 years do here. Adolescence is known as a time of intense change in social relationships in typical participants (Foulkes and Blakemore 2016) and those with autism (Adreon and Stella 2001; Carrington et al. 2003). For example, hypersensitivity to social rejection can induce strong anxiety in typical adolescents, altering their behaviour (Perino et al. 2016), and social anxiety is also common in people with ASC (Bejerot et al. 2014). In an evaluation of 8-15 year old high functioning people with ASC, it was reported that behavioural avoidance and social anxiety may increase with age in ASC group compared to the healthy controls (Kuusikko et al. 2008). This implies that the preference for non-social stimuli seen in the older ASC participants here and in the adult sample of Dubey et al (2015) may partly reflect late developing social anxiety, rather than an intrinsic and early-emerging difference in social motivation. However, larger scale studies over a wider age range and with concurrent measures of social anxiety would be needed to confirm this.

**Supplementary Section 8**

**Effect of age on social seeking in typical adolescents**

The brain undergoes major changes during this adolescence, the lag between the development of the reward system and the prefrontal cortex might result in odd decision making in this age group (Blakemore and Robbins 2012). Bjork et al (2004) compared groups of 12-17 years and 22-28 years olds typical participants on a Monetary Incentive Delay task. The results showed that the adolescent group had comparatively reduced activation in Ventral Striatum (related to motivation) for anticipation of monetary reward but they were not different from adults in brain activation for consumption (final presentation) of these reward. These results indicate that though typical adolescents may not have lower liking for the rewarding stimuli than adults, they may not make same effort to seek it as adults do. Similar conclusions were drawn from the behavioural studies by Kohls et al (2009) and Demurie et al (2012) who used the Social Incentive Delay task with typical adolescents (between 8-12 and 8-16 years respectively). Both these studies reported that the social rewards did not improve the task performance for this group. Demurie et al (2012) further reported that adolescent participants showed higher liking for social stimuli but there was no positive relation with their reaction time while anticipating social rewards. All these results may indicate that even though adolescents may prefer social over non-social stimuli they may not make higher effort to seek it, which may result in the lack of a stimuli preference as seen in the present study.

**References**

Adreon, D., & Stella, J. (2001). Transition to Middle and High School: Increasing the Success of Students with Asperger Syndrome. *Intervention in School and Clinic*, *36*(5), 266–271. doi:10.1177/105345120103600502

Aldridge, F. J., Gibbs, V. M., Schmidhofer, K., & Williams, M. (2012). Investigating the Clinical Usefulness of the Social Responsiveness Scale (SRS) in a Tertiary Level, Autism Spectrum Disorder Specific Assessment Clinic. *Journal of Autism and Developmental Disorders*, *42*(2), 294–300. doi:10.1007/s10803-011-1242-9

Bejerot, S., Eriksson, J. M., & Mörtberg, E. (2014). Social anxiety in adult autism spectrum disorder. *Psychiatry Research*, *220*(1-2), 705–707. doi:10.1016/j.psychres.2014.08.030

Bjork, J. M., Knutson, B., Fong, G. W., Caggiano, D. M., Bennett, S. M., & Hommer, D. W. (2004). Incentive-Elicited Brain Activation in Adolescents: Similarities and Differences from Young Adults. *Journal of Neuroscience*, *24*(8), 1793–1802. doi:10.1523/JNEUROSCI.4862-03.2004

Blakemore, S.-J., & Robbins, T. W. (2012). Decision-making in the adolescent brain. *Nature Neuroscience*, *15*(9), 1184–1191. doi:10.1038/nn.3177

Bölte, S., Westerwald, E., Holtmann, M., Freitag, C., & Poustka, F. (2011). Autistic Traits and Autism Spectrum Disorders: The Clinical Validity of Two Measures Presuming a Continuum of Social Communication Skills. *Journal of Autism and Developmental Disorders, 41*(1), 66–72. doi:10.1007/s10803-010-1024-9

Carrington, S., Papinczak, T., & Templeton, E. (2003). A phenomenological study: The social world of five adolescents who have Asperger’s syndrome. *Australian Journal of Learning Disabilities*, *8*(3), 15–20. doi:10.1080/19404150309546734

Constantino, J. N., & Gruber, C. P. (2005). *Social Responsiveness Scale^TM^ (SRS^TM^)* (2nd ed.). Los Angeles: Western Psychological Services.

Demurie, E., Roeyers, H., Baeyens, D., & Sonuga-Barke, E. (2012). The effects of monetary and social rewards on task performance in children and adolescents: Liking is not enough. *International Journal of Methods in Psychiatric Research*, *21*(4), 301–310. doi:10.1002/mpr.1370

Dubey, I., Ropar, D., & Hamilton, A. F. de C. (2015). Measuring the value of social engagement in adults with and without autism. *Molecular Autism*, *6*, 35. doi:10.1186/s13229-015-0031-2

Dunn, Dunn, Whetton, & Burley. (1997). *British Picture Vocabulary Scale - Third Edition (BPVS-3)* (3rd ed.). GL assessment.

Foulkes, L., & Blakemore, S.-J. (2016). Is there heightened sensitivity to social reward in adolescence? *Current Opinion in Neurobiology*, *40*, 81–85. doi:10.1016/j.conb.2016.06.016

Lord, C. , Rutter, M., & Couteur, A. Le. (1994). The Autism Diagnostic Interview-Revised: A revised version of a diagnostic interview for caregivers of individuals with possible pervasive developmental disorders. *Journal of Autism and Developmental Disorders* 24(5), 659–685. doi:10.1007/BF02172145

Lord, C., Risi, S., Lambrecht, L., Cook Jr, E. H., Levelthal, L. B., DiLavore, C. P., Pickles, A. & Rutter, M. (2000). The Autism Diagnostic Observation Schedule—Generic: A Standard Measure of Social and Communication Deficits Associated with the Spectrum of Autism. *Journal of Autism and Developmental Disorders*, *30*(3), 205-223. doi:10.1023/A:1005592401947

Kohls, G., Peltzer, J., Herpertz-Dahlmann, B., & Konrad, K. (2009). Differential effects of social and non-social reward on response inhibition in children and adolescents. *Developmental science*, *12*(4), 614–25. doi:10.1111/j.1467-7687.2009.00816.x

Kuusikko, S., Pollock-Wurman, R., Jussila, K., Carter, A. S., Mattila, M.-L., Ebeling, H., et al. (2008). Social Anxiety in High-functioning Children and Adolescents with Autism and Asperger Syndrome. *Journal of Autism and Developmental Disorders*, *38*(9), 1697–1709. doi:10.1007/s10803-008-0555-9

Liddle, E. B., Batty, M. J., & Goodman, R. (2009). The Social Aptitudes Scale: an initial validation. *Social Psychiatry and Psychiatric Epidemiology*, *44*(6), 508–513. doi:10.1007/s00127-008-0456-4

Perino, M. T., Miernicki, M. E., & Telzer, E. H. (2016). Letting the good times roll: adolescence as a period of reduced inhibition to appetitive social cues. *Social Cognitive and Affective Neuroscience*, nsw096. doi:10.1093/scan/nsw096

Raven, J. C., Court, J. H., & Raven, J. C. (2003). *Raven’s Coloured Progressive Matrices*. Peason Education.
